# Occipital osteomylelitis and epidural abscess after occipital nerve block: A case report

**DOI:** 10.1080/24740527.2017.1360725

**Published:** 2018-02-27

**Authors:** Sean D. Christie, Nelofar Kureshi, Ian Beauprie, Renn O. Holness

**Affiliations:** a Division of Neurosurgery, Dalhousie University/QEII Health Sciences Centre, Halifax, NS, Canada; b Department of Anesthesia, Pain Management & Perioperative Medicine, Dalhousie University/QEII Health Sciences Centre, Halifax, NS, Canada; c Cornwall Regional Hospital, University of the West Indies, The University of the West Indies, Mona, Jamaica

**Keywords:** epidural abscess, occipital nerve block, occipital neuralgia

## Abstract

Occipital neuralgia is a paroxysmal jabbing pain in the distribution of the greater or lesser occipital nerves accompanied by diminished sensation in the affected area. Occipital nerve block is a common diagnostic and therapeutic tool used in the course of occipital neuralgia and is considered a safe treatment with few localized adverse events. Occipital nerve block is also indicated for cervicogenic and cluster headache and is often used as a rescue treatment for headaches not responding to conventional therapies. We describe a case of epidural abscess formation 16 days following occipital nerve block in a patient with no underlying medical conditions. This case report emphasizes the importance of strict aseptic technique to reduce infection rates in patients undergoing this procedure, despite the overall safety of occipital nerve block. Clinicians must remain aware of acute and late complications arising postprocedure for the safe practice of this technique.

## RÉSUMÉ

La névralgie occipitale est une douleur lancinante paroxystique dans la distribution des nerfs du grand ou du petit occipital qui s’accompagne d’une diminution des sensations dans la zone affectée. Le bloc du nerf occipital, un outil diagnostique et thérapeutique communément utilisé dans le cadre d’une névralgie occipitale, est considéré comme un traitement sécuritaire qui n’entraine que peu d’effets indésirables localisés. Le bloc du nerf occipital, également indiqué pour traiter la céphalée cervicogénique et la céphalée vasculaire de Horton, est souvent utilisé en tant que traitement de secours pour les céphalées qui ne répondent pas aux thérapies conventionnelles. Nous décrivons un cas de formation d’un abcès épidural 16 jours après le bloc du nerf occipital chez un patient sans affection médicale sous-jacente. L’étude de cas met l’accent sur l’importance d’une stricte conformité aux techniques d’asepsie afin de réduire les taux d’infection chez les patients soumis à cette procédure, malgré le caractère sécuritaire du bloc du nerf occipital. Les cliniciens doivent demeurer vigilants quant aux complications aigues et tardives qui peuvent survenir après la procédure afin d’appliquer cette technique de manière sécuritaire.

## Introduction

Occipital neuralgia was first described by Beruto Lentijo and Ramos in 1821. The International Headache Society defines occipital neuralgia as a paroxysmal jabbing pain in the distribution of the greater or lesser occipital nerves accompanied by diminished sensation or dysesthesia in the affected area.^1^ This is often accompanied by tenderness over the affected nerve. Occipital neuralgia is considered to be a subset of cervicogenic headache, in which there is unilateral, occipitofrontal head pain associated with changes in neck position or pressure and tenderness of cervical and paraspinal tissues.^2,3^ Although most patients have idiopathic occipital neuralgia, a wide array of causes secondary to structural nerve injury have been identified. These include congenital malformations, neoplasms of C2 and C3 nerve roots, degenerative disease of the spine, and disorder of the peripheral nervous system. Trauma or compression of greater and/or lesser occipital by degenerative cervical spine changes also contributes to the etiology of occipital pain.

Occipital nerve block is indicated for cervicogenic headache, cluster headache, and occipital neuralgia. Although it is not a proven indication for migraine, several observational studies demonstrate that it may be used as a rescue treatment for headaches not responding to conventional therapies or as an adjunctive treatment for medication overuse in headache.^4^ Temporary response to nerve block may last from days and weeks to several months and forms part of the diagnostic criteria for occipital neuralgia.^5^ The procedure is performed with anatomic landmark or ultrasound guidance and there are two different techniques for ultrasound-guided greater occipital nerve block. The proximal/central technique is performed at the level of the second cervical vertebra and has the potential for a higher incidence of complications, including epidural abscess, in comparison to the peripheral/distal technique that is performed at the level of the nuchal ridge and is used more commonly.^6,7^ Short- or long-acting local anesthetics alone or in combination with a corticosteroid may be injected. Commonly used anesthetics in occipital nerve block are lidocaine, bupivacaine, and mepivacaine.^8^ No major complications from this procedure have been previously reported.

## Case report

A 63-year-old man presented to his community hospital with headache, fever, and confusion. Earlier that morning, his wife had been awakened from sleep at approximately 5 a.m., finding her husband thrashing and shaking in bed. The patient was soaked with sweat, incontinent of urine, warm to touch, and very confused. Upon arrival at the local emergency department, he was found to be in generalized sepsis. His heart rate was 120, his blood pressure was 136/76 mmHg, and his temperature was 38.5°C. Initial blood work revealed a white blood cell count of 15.8 × 10^9^/L. Chest x-ray and urinalysis were normal. Over the next few hours in the emergency department, the patient became hypotensive with systolic blood pressures fluctuating between 75 and 90 mmHg. He was treated with intravenous fluids and antibiotics and transferred to the intensive care unit of the community hospital.

The patient’s medical history revealed that he had been suffering with left-sided occipital region headaches for more than 5 weeks. His primary care physician had seen him several times for this complaint and had made the diagnosis of occipital neuralgia. When his symptoms failed to respond to analgesics and gabapentin, local nerve blocks were used. The patient had received two occipital nerve blocks using methylprednisolone and xylocaine; the two sessions were separated by 11 days. After this, the patient continued to have the headaches, which worsened in severity. He also began to develop a tender swelling in the area where the nerve blocks had been performed. This swelling continued to grow in size and tenderness. The patient developed initial symptoms of epidural abscess 16 days after receiving the last occipital nerve block.

Upon admission to the intensive care unit and resuscitation with intraenous (IV) fluids, cloxacillin, and ceftriaxone, the patient’s hemodynamics stabilized and he became afebrile. Given the history, he underwent a computed tomography scan of his head, which showed a subcutaneous fluid collection and underlying epidural collection in the region over the left posterior fossa (Figure 1a). There was also evident erosion of the bony skull between the two fluid collections (Figure 1b). Magnetic resonance imaging was performed, which confirmed the diagnosis of subcutaneous and epidural abscess with focal osteomyelitis of the skull (Figures 2a, 2b, and 2c). The following day, he was transferred from the community hospital to neurosurgical services at the tertiary care center.

**Figure 1a.**
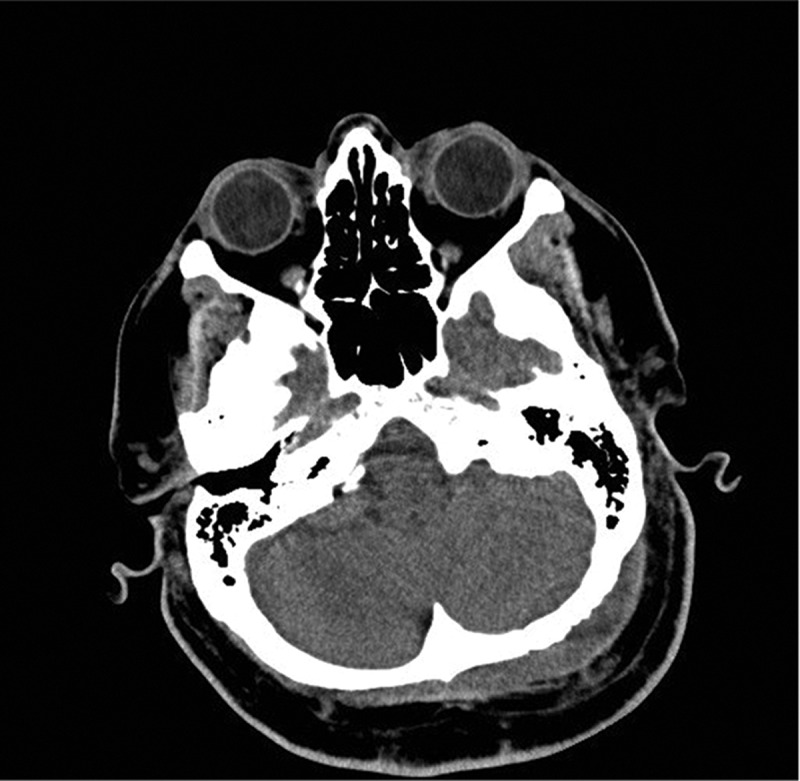
CT scan showing soft tissue swelling suggestive of epidural abscess of left posterior fossa.

**Figure 1b.**
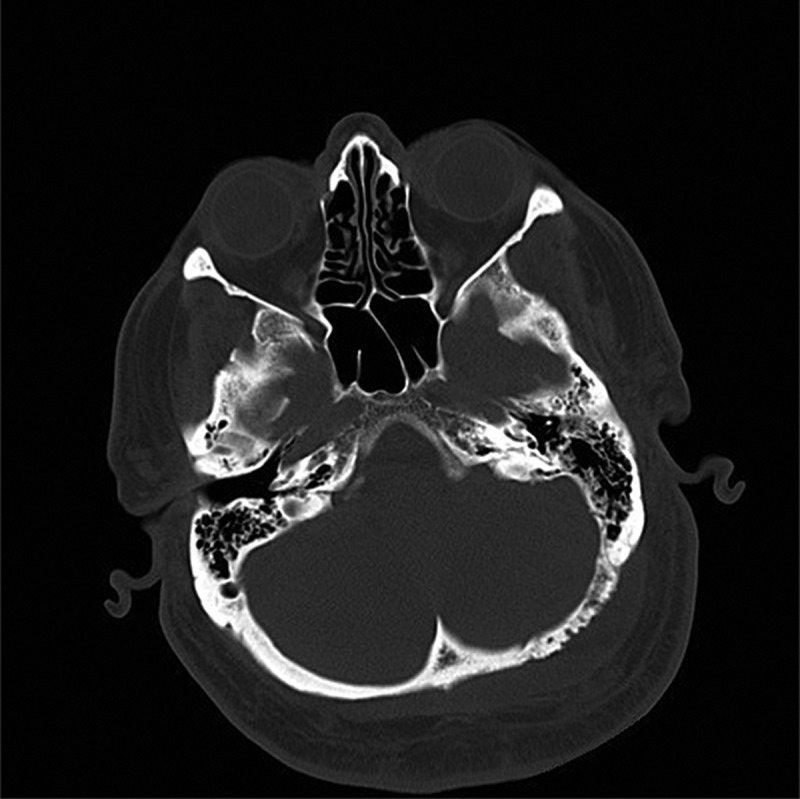
CT scan showing mottled appearance of left occipital bone, consistent with osteomyelitis.

**Figure 2a.**
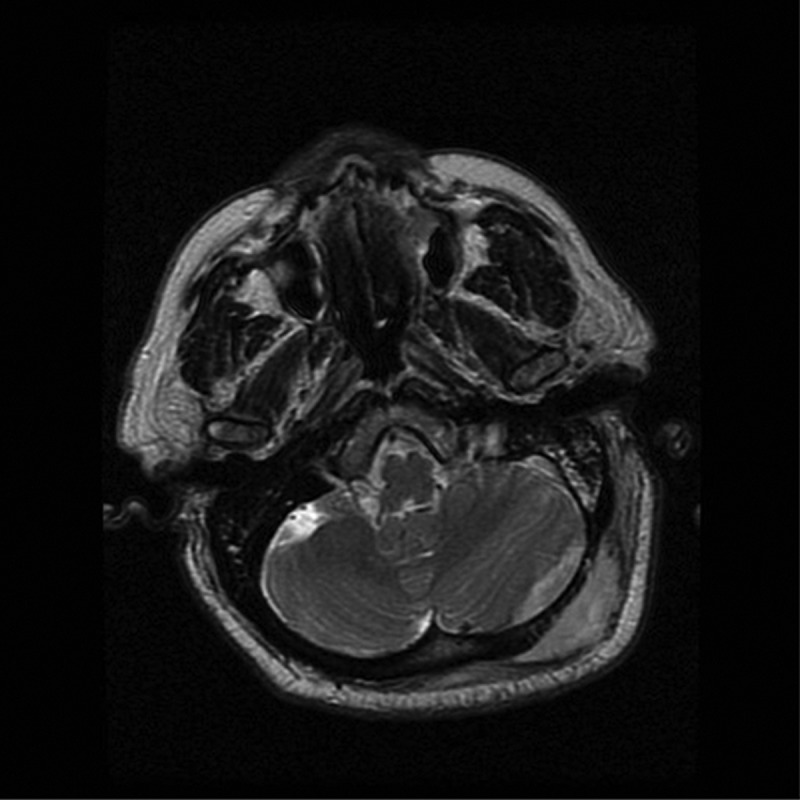
MRI axial T2 image revealing high signal intensity within left suboccipital soft tissue and epidural space.

**Figure 2b.**
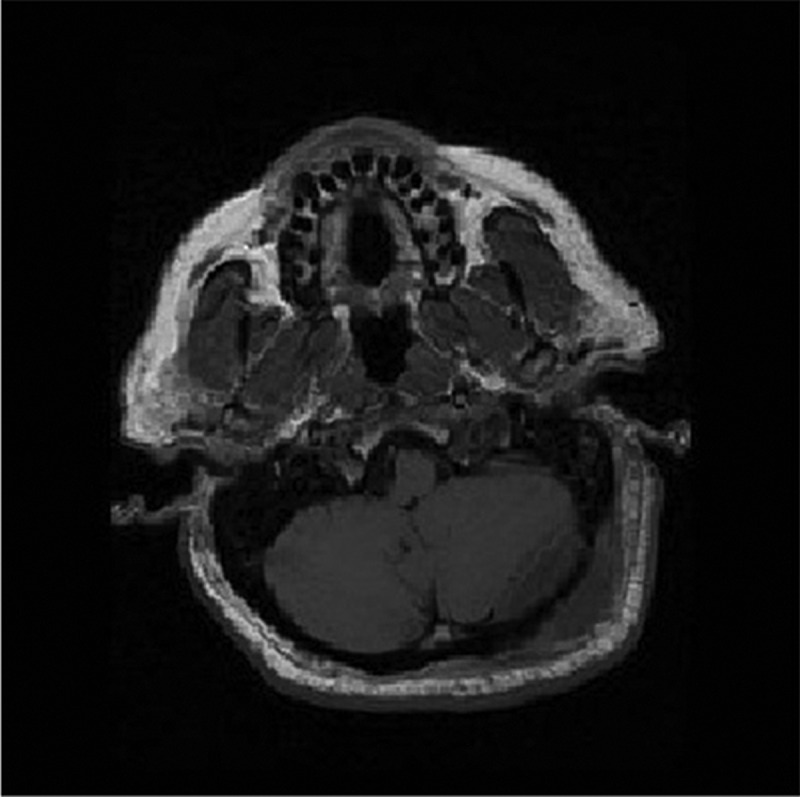
T1 axial unenhanced image showing left suboccipital and epidural low signal intensity suggestive of soft tissue swelling.

**Figure 2c.**
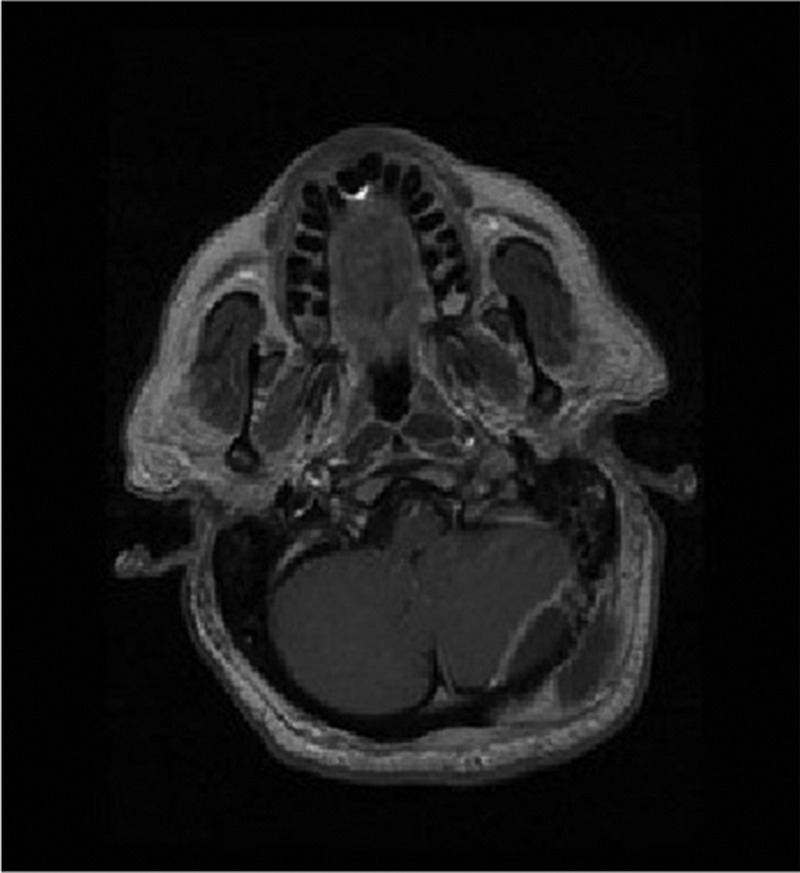
TI axial post gadolinium image demonstrating a ring-enhancing lesion of left suboccipital soft tissue and posterior fossa epidural space consistent with abscess.

Upon admission, the patient was alert and oriented with a heart rate of 95, blood pressure of 150/80 mmHg, and temperature of 38.1°C. Motor, sensory, and cerebellar reflex examinations as well cranial nerve assessment were normal. Head and neck assessment noted a boggy fluctuant tender region over the left occipital area with no erythema. There was no evidence of meningismus. The patient was taken to the operating room for incision and drainage of the subcutaneous and epidural abscess. Frank pus was extruded from both the subcutaneous and epidural cavities. A small local craniectomy was performed to remove the infected bone and a Hemovac drain was placed subcutaneously into the epidural space and removed 2 days postoperatively. The patient was continued on IV antibiotics and had an uneventful early postoperative recovery. Cultures from the abscess grew *Streptococcus milleri* species. A consulting infectious disease specialist recommended IV ceftriaxone (2gm IV q 12h), which was continued for a period 4 weeks, and the patient subsequently made a complete recovery.

## Discussion

Occipital neuralgia has a presentation similar to that of other types of headache, and because of its challenging diagnosis, a reliable incidence and prevalence of this condition remains unknown.^9^ The most common cause of occipital neuralgia is greater occipital nerve irritation (90% of cases); 8.7% cases involve both greater and lesser occipital nerves.^10^

Occipital nerve block is a common diagnostic and therapeutic tool used in the course of occipital neuralgia, and injections are commonly performed by both family physicians and specialists. Ultrasound-guided approaches are superior to landmark techniques for favorable outcome with occipital nerve block.^11^ The greater occipital nerve (GON) provides cutaneous innervation to the posterior scalp, whereas the lesser occipital nerve supplies scalp sensation lateral to the GON to the posterior auricle. In patients with occipital neuralgia, the GON may be blocked alone or with the lesser occipital nerve for peripheral nerve block. Local anesthetics including lidocaine, mepivacaine, and bupivacaine may be injected as monotherapy or as combinations. Corticosteroids may be added for patients who do not respond to infiltration with local anesthetic only.

In general, occipital nerve block is considered a safe treatment with few localized adverse events, such as transient injection site pain, hematoma, dizziness and lightheadedness.^12^ Unwanted cosmetic side effects such as alopecia and cutaneous atrophy are also associated with use of local corticosteroids in the GON region. Caution is advised in individuals with previous cranial surgeries and skull defects, because loss of consciousness may occur with anesthetic infiltration into the nervous system. Systematic toxicity from absorption of local anesthetics is a rare complication but may lead to seizures and cardiac conduction defects.^13^ Postprocedural infections may occur with microorganisms such as *Staphylococcus aureus*, coagulase-negative *Staphylococcus, Enterococcus, Escherichia coli, Pseudomonas*, and miscellaneous aerobic gram-negative bacilli. Infections following nerve blocks are most commonly associated with in-dweliing catheters; 1%–7% of patients develop local infection following insertion of regional anesthesia catheters.^14^ Specific infection rates following occipital nerve block for occipital neuralgia have not been reported in the literature. Other risk factors for infection include severe diabetes mellitus, immunosuppression (secondary to chemotherapy or systemic steroid therapy), dialysis, smoking, alcoholism, and drug abuse.

Epidural abscess caused by *Streptococcus milleri* group is uncommon in the literature. *Streptococcus milleri* occurs naturally in the flora of the oral cavity, gastrointestinal tract, and genitourinary tract. It commonly causes infections at remote sites such as lung, heart, liver, and brain by hematogenous spread, although its implication in epidural abscess is rare.^15^ The first report of *Streptococcus milleri* spinal epidural abscess was documented in a healthy young pregnant woman who had no underlying medical conditions or evident risk factors for invasive disease.^16^ A second report described the case of a 56-year-old female who developed lumbar septic arthritis, epidural abscess, bilateral iliopsoas, and subcutaneous abscesses due to *Streptococcus milleri* infection after receiving lower back and gluteal acupuncture for relief of musculoskeletal back pain.^17^ In reports of brain abscess caused by *Streptococcus milleri*, paranasal sinuses, dental, facial soft tissue, deep neck space, and peritonsillar regions have been identified as sites of infection.^18^ It is possible that our patient had oral or gastrointestinal bacteremia that led to seeding of the epidural space.

Our patient received a mixture of steroid and local anesthetic for both occipital nerve blocks. Whether steroids provide an added benefit over local anesthetics in greater occipital nerve block and trigger point injections is a controversial topic. Previous studies have shown that the adding steroids for greater occipital nerve blocks is not superior to the anesthetic drug alone (2% lidocaine and 0.5% bupivacaine + either saline or 40 mg triamcinolone)^19^ and nerve blocks with steroids (0.5% bupivacaine plus 20 mg methylprednisolone) are not superior to placebo (normal saline plus 1% lidocaine).^20^ In contrast, Ambrosini et al.^21^ found a significant difference in the response of cluster headaches to local anesthetic with steroid (lidocaine with betamethasone) versus anesthetic alone. Local steroid injection may be a contributing factor to the patient’s subcutaneous and epidural abscess formation.

It is also possible that the patient’s infection was due to a lack of aseptic technique from previous occipital nerve blocks. The American Society of Anesthesiologists advocates strict adherence to aseptic techniques to avoid contamination of sterile injection equipment and reduce health care–associated infections. Aseptic technique prevents harmful microorganisms on hands, surfaces, or equipment from being introduced to the injection site. Hands must be cleaned before wearing and after removal of sterile gloves. Pre-injection skin preparation with an antiseptic solution is a clinical necessity for the administration of regional anesthesia and is critical for the prevention of surgical site infection.^22^ Prior to the procedure, skin preparation using chlorhexidine/alcohol solutions can reduce the risk of infection by lowering the risk of contamination from the patient’s own skin flora. The antiseptic solution should be allowed to dry before commencing the procedure for maximal benefit. Single-dose vials of drug must not be used for multiple patients and a new sterile syringe should be used each time any medication or solution is accessed.^23^

## Conclusion

To our knowledge, this is the first report of epidural abscess subsequent to occipital nerve block for occipital neuralgia in a patient with no underlying medical conditions. Although occipital nerve block is a popular diagnostic and treatment modality for occipital headache, it was not associated with success in relieving our patient’s headache. This case report emphasizes the importance of strict aseptic technique to reduce infection rates in patients undergoing this procedure, despite the overall safety of occipital nerve block. Awareness of acute and late complications arising postprocedure is critical for the safe clinical practice of this technique.

## Disclosure of Interest

Sean Christie is a consultant for Medtronic Canada (spine). Nelofar Kureshi has no conflicts of interest to declare. Ian Beauprie has no conflicts of interest to declare. Renn Holness has no conflicts of interest to declare.
